# Reconstruction of freehand 3D ultrasound based on kernel regression

**DOI:** 10.1186/1475-925X-13-124

**Published:** 2014-08-28

**Authors:** Xiankang Chen, Tiexiang Wen, Xingmin Li, Wenjian Qin, Donglai Lan, Weizhou Pan, Jia Gu

**Affiliations:** Shenzhen Institutes of Advanced Technology, Chinese Academy of Sciences, 518055 Shenzhen, China; The Shenzhen Key Laboratory for Low-cost Healthcare, 518055 Shenzhen, China; School of Computer, South China Normal University, 510631 Guangzhou, China

**Keywords:** Freehand ultrasound, Kernel regression, Reconstruction, Interpolation, Nonparametric statistics

## Abstract

**Introduction:**

Freehand three-dimensional (3D) ultrasound has the advantages of flexibility for allowing clinicians to manipulate the ultrasound probe over the examined body surface with less constraint in comparison with other scanning protocols. Thus it is widely used in clinical diagnose and image-guided surgery. However, as the data scanning of freehand–style is subjective, the collected B-scan images are usually irregular and highly sparse. One of the key procedures in freehand ultrasound imaging system is the volume reconstruction, which plays an important role in improving the reconstructed image quality.

**System and methods:**

A novel freehand 3D ultrasound volume reconstruction method based on kernel regression model is proposed in this paper. Our method consists of two steps: bin-filling and regression. Firstly, the bin-filling step is used to map each pixel in the sampled B-scan images to its corresponding voxel in the reconstructed volume data. Secondly, the regression step is used to make the nonparametric estimation for the whole volume data from the previous sampled sparse data. The kernel penalizes distance away from the current approximation center within a local neighborhood.

**Experiments and results:**

To evaluate the quality and performance of our proposed kernel regression algorithm for freehand 3D ultrasound reconstruction, a phantom and an in-vivo liver organ of human subject are scanned with our freehand 3D ultrasound imaging system. Root mean square error (RMSE) is used for the quantitative evaluation. Both of the qualitative and quantitative experimental results demonstrate that our method can reconstruct image with less artifacts and higher quality.

**Conclusion:**

The proposed kernel regression based reconstruction method is capable of constructing volume data with improved accuracy from irregularly sampled sparse data for freehand 3D ultrasound imaging system.

## Introduction

Image-guided technique has been widely used in minimally invasive surgery which can greatly reduce the patient’s pain, improve the operation success rate and accelerate postoperative recovery. Compared with computed tomography (CT) imaging and magnetic resonance imaging (MRI), ultrasound is more suitable in intraoperative imaging for its real-time imaging advantage. Further more, ultrasound is non-invasive, non-ionizing, portable, and low-cost.

As 2D ultrasound cannot provide complete volume data of tissues and organs, 3D ultrasound imaging system is put forward to overcome such limitations by constructing abundant 3D data. A number of approaches for constructing 3D ultrasound volume data have been proposed, which can be grouped into three categories: dedicated 3D probes, mechanical scanning and freehand scanning [1]. 3D probes relies on an sophisticated mechanism for scanning a predefined region of interested (ROI), and can provide 3D volume data in real-time. Nevertheless they are expensive and have limitation on scanning large organs [2]. The mechanical scanning is usually formed of a scanning head as well as a stepping motor, which move and rotate the 2D transducer and synchronously record positions and orientations data [3]. Unfortunately, the scanning range is still limited [2]. In comparison with the above approaches, freehand 3D ultrasound is relatively low-cost and flexible. The irregularly sampled B-scan images with corresponding positions and orientations are scanned and recorded by a 2D probe with transducer, which can be manipulated freely to view the desired anatomical section.

A number of algorithms for reconstructing 3D ultrasound images from these irregularly 2D B-scan images. These algorithms fall into three groups: Voxel-Based Methods (VBM), Pixel-Based Methods (PBM) and Function-Based Methods (FBM) [4]. VBMs traverse all voxels in a target volume and inserts corresponding pixels from the input images. An usual method of implementation this way is the Voxel Nearest Neighbor (VNN) [5]. VNN traverses each volume voxel and assigns the value of nearest image pixel. VNN method can preserve the most original texture patterns from B-scan images, but it also has a trend to generate large reconstruction artifacts when the distance of the voxel to the B-scan image plane is large. Another algorithm is the Distance-Weighted (DW) [6]. DW traverses each volume voxel and assigns the value of average its local neighborhood pixels weighted by the inverse distances between those pixels and the voxel center. DW method trends to averaging, so it has the advantage of suppress speckle noise, however, it also smooth out the 3D image boundaries. In [7], Huang et al. introduce a median-filter-based reconstruction method to improve the quality of volume reconstruction by utilizing median filter to reduce speckle noise. In [2, 8–10], Huang et al. propose some algorithms by improving the DW algorithm. Such as the recently present a adaptive Gaussian distance weighted (AGDW) method to improve the quality of reconstructed image. The AGDW evaluates the homogeneity of the neighborhood for each voxel according to the local variance/mean of neighboring pixels. If a voxel is deemed as a homogeneous region, its neighboring pixels are averaged. Otherwise, the voxel value is calculated using an adaptive Gaussian distance weighted method with respect to the local statistics. However, the AGDW method trends to over-emphasize the effect of speckle reduction, the patch-like pattern can be observed from their experimental results. The Probe Trajectory (PT) method [11] builds on the DW algorithm. Instead of using the orthogonal projection of points to the nearest B-scans, a probe trajectory is estimated and used for finding the corresponding pixels in the nearest B-scans. PBMs traverse the input pixels and insert them into the corresponding target volume voxels. it consist of two steps: a Distribution Step (DS) and a Hole-Filling Step (HFS). An usual method of implementation this way is the Pixel Nearest Neighbor (PNN). In the DS, PNN traverses each pixel in all the 2D Ultrasound images, each pixel value is filled into the nearest voxel (Distribution Step). if multiple pixels contribute to the same voxel, the averaged value [12, 13], the maximum value [12], the most recent value [14] or the first value [15] are usually selected for assigned it. In the HFS, the volume from the DS has some empty voxels, each empty voxel is filled with the nearby already filled voxels. The usual method for this purpose is average or maximum [12, 16] or a median [16] of those voxels. Dewi et al. [17] propose a Hole-filling algorithm by improving the Olymplic operator. The algorithm shows a better empty voxel estimation than conventional Olympic, Average, and Maximum. The PNN method causes great blurring as well as relatively large reconstruction error. In [18], a method named direct frame interpolation (DFI) method is presented to creates additional intermediate image frames by directly interpolating between two or more adjacent image frames of the original image series. Then the DFI method fills the the target volume using the original frames in combination with the additionally constructed frames. The DFI method requires the sampled B-scan slices not to be intersected. However, the intersection among the B-scan slices could not be avoided in the freehand scanning configuration. In [3], a method based on Cyclic Regularized Savitzky-Golay (CRSG) filter is introduced to reconstruct freehand 3D ultrasound volume with mechanical linear scanning sweep. However, such parallel scanning sweep is usually not available for the freehand scanning. To preserve the detail of boundaries, An Fast Marching Method (FMM) algorithm has been recently proposed by [1], it consists of DS and HFS. In DS, it similar to DS of PNN, In HFS, it advances the interpolation boundary along its normal direction and fills the area closest to known voxel points in first. it improvement in reconstruction accuracy and efficiency. FBMs utilize estimate functions by taking use of the input data for creating the voxel grid. Rohling et al. [6] propose the Radial Basis Function (RBF) interpolation is an approximation with splines that tries to use the underlying shape of the data in the volume reconstruction. Overfitting phenomenon is usually unavoidable for the Spline method. Sanches and Marques [19] present the Rayleigh reconstruction/interpolation with a Bayesian framework estimates a function for the tissue by statistical methods. The Rayleigh method also trend to suppress spckle noise but smooth out the boundaries.

This paper develops a new freehand 3D ultrasound reconstruction algorithm based on kernel regression (KR) aims to suppress speckle noise and preserve boundaries. its corresponding ideas come from nonparametric estimation [20]. Nonparametric estimation has various applications, such as 2D image denoising, upscaling, interpolation and fusion [21], feature extraction for 3D MR image denoising [22]. In the following, we demonstrate the details of KR applied in freehand 3D ultrasound reconstruction.

## System and methods

### System

**Figure 1:**
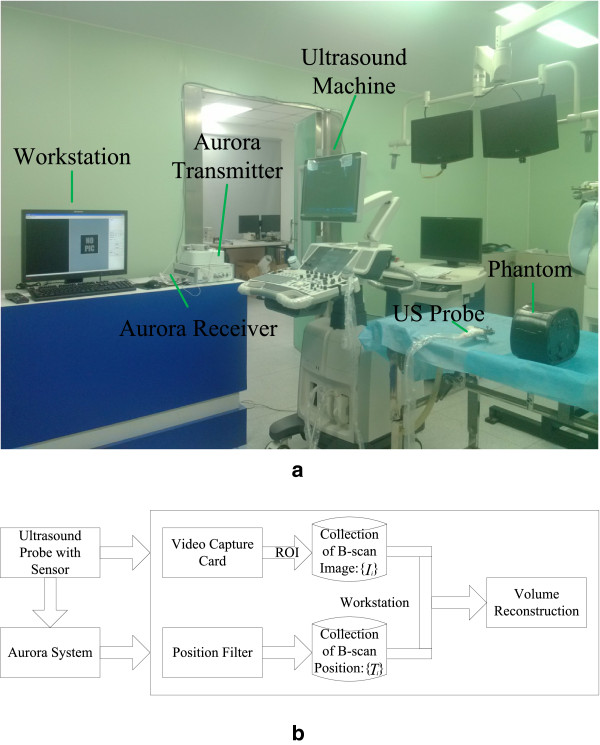
**Freehand 3D ultrasound system. (a)** The main components of our system. **(b)** The framework of our system.

The freehand 3D ultrasound system consists of three modules: a conventional 2D ultrasound scanner (DC-7, Mindray Medical International Ltd., Shenzhen, China) used to acquire ultrasound image, an electromagnetic spatial sensing device (Aurora [23], NDI, Ontario, Cannada) acquiring the position and orientation of ultrasound images and surgical instruments, and a workstation with custom-designed software used for data collection, volume reconstruction, and visualization [1]. Figure 1 illustrate this system framework. The spatial information (position (*x*,*y*,*z*) and orientation (*R*_*x*_,*R*_*y*_,*R*_*z*_)) of ultrasonic probe and surgical instrument embeding sensor is recorded by Aurora system connects to the workstation through its USB port so that it can be acquired by a custom-designed software by use of Aurora system API. Besides, the real-time ultrasound video is captured by a video capture card (RGB-133, VTimage Inc., Shenzhen, China) installed in the workstation.

### Data collection

Before volume reconstruction, data collection is a very important step which influences accuracy and efficiency of reconstruction. Data collection consists of two steps: calibration between spatial data and 2D B-scan images, and selecting ROI (region of interest). In this paper, the set of B-scan image *I*_*i*_ and its position *T*_*i*_ are collected by the data collection method of [1].

### Volume reconstruction

A 3D volume data is reconstructed from the collected data, including the 2D B-scan images and its spatial information. In this study, the algorithm for volume reconstruction is composed of two stages: bin-filling and regression.

#### Bin-filling stage

The bin-filling stage is to map the pixel in 2D B-scans into the voxel in 3D volume data based on its corresponding positional information. In this freehand 3D ultrasound system, the mapping of the coordinate system from the 2D B-scans to 3D volume is named after forward mapping and is defined as
1

where *V*_*p*_ is the physical position, *M* is the forward transformation matrix, and *V*_*r*_ is the resulting voxel location in the reconstructed 3D volume data. The forward transformation matrix *M* must be decomposed and implemented to find an matrix for this transformation, which is discussed in detail in [1].

#### Regression stage

**Figure 2:**
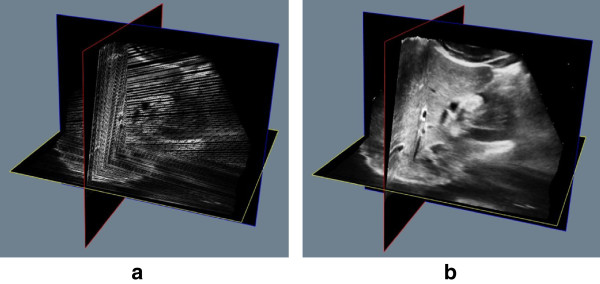
**A liver of human volumn data with our method. (a)** The bin-filled result. **(b)** The reconstructed result.

Since the scanning of freehand-style is subjective, the collected B-scan images are usually irregular and highly sparse. Therefore, there are some gaps in the reconstructed volume after the bin-filling stage, as addressed in Figure 2(a). The goal of the regression stage is to make the nonparametric estimation [20] for the whole volume data from the previous sparse volume data.

The sparse volume data after the bin-filling stage are given by
2

where *r*(·) is the regression function, *X*_*i*_ = (*X*_*i*1_,*X*_*i*2_,*X*_*i*3_)^*T*^ is the three-dimension coordinate of data, *ε*_*i*_s are the independent and identically distributed zero mean noise values.

Specifically, if *X* is near the sample at *X*_*i*_, we can approximate it with a *N*-term Taylor series
3

A least-squares formulation capturing this idea is to solve the folowing optimization problem:
4

where *K*(·) is the kernel function which penalizes distance away from the local position where the approximation is centered, and the smoothing parameter *h* (bandwidth) controls the strength of this penalty. In particular, the function *K* is a symmetric function which attains its maximum at zero, satisfying
5

where *c* is a constant value. The choice of the particular form of the function *K* is usually Gaussian, exponential, or other forms, which comply with the above constraints. Because the choice of the kernel has little impact on the accuracy of estimation. Therefore, the Gaussian kernel, being computational complexity, is selected in this paper.

For the estimation problem based on Least Square Method upon showed in Equation 4, the order *N* effect the accuaracy and complexity of local approximation of the volume data. Therefore, it must be appropriately chosen. In the nonparametric statistics literature, locally constant, linear, and quadratic approximations (corresponding to *N*=0,1,2) have been considered most widely [24–27].

The Kernel function *K* is now a function of 3 variables. Given a nonsingular positive definite 3 × 3 bandwidth matrix *H*, which is defined
6

Often, one scales each covariate to have the same mean and variance, then use the kernel
7

where *K* is any one-dimensional kernel. therefore, there is a single bandwidth parameter *h*. At a target value *X* = (*X*_1_,*X*_2_,*X*_3_)^*T*^, the local sum of squares is given by
8

where
9

The estimator is
10

where  is the value of *β* = (*β*_0_,⋯,*β*_3_)^*T*^ that minimizes the weighted sums of squares. The solution  is
11

where
12

and *W*_*x*_ is the diagonal matrix whoses (*i*,*i*) element is *w*_*i*_(*X*). Therefore,
13

where *e*_1_ is a column vector with all elements equal to zero but the first element equal to one,  is the final values of reconstructed volume data.

## Experiments and results

To evaluate the quality and performance of our proposed KR algorithm for freehand 3D ultrasound reconstruction, a phantom and an in-vivo liver organ of human subject are scanned with our freehand 3D reconstruction system. The parameters of the order *N*, the size of kernel *R* and the bandwidth *h* of KR are assigned with 1, 15 and 0.5 respectively. The proposed method is compared with the classical VNN, PNN, Spline and PT methods and the recent FMM method. The accuracy of the reconstruction results is evaluated via root mean square error (RMSE). The running time of different methods on all scanned datasets are given for their performance and efficiency comparison. All of the methods are implemented in C++, and the computer is equipped with a Intel Core i3-2120 3.3 GHz and 2 GB RAM.

### Qualitative results

**Figure 3:**
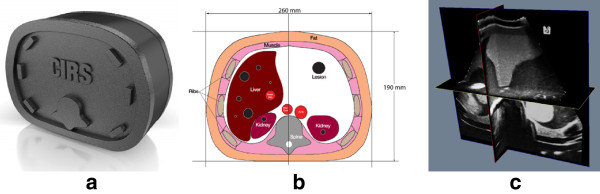
**A 3D ultrasonic abdominal phantom with our method. (a)** The outer structure of phantom. **(b)** The inner structure of phantom. **(c)** The reconstructed volumn data of phantom with our method.

The first experiment is conducted on an abdominal phantom (CIRS Model 057), which is made from proprietary materials to accurately mimic human tissues under MRI, CT, and ultrasound. The phantom mainly contains simulated lungs, liver, hepatic vesels, ribs, vertebra, kidneys, and abdominal aorta. The freehand scan of the phantom is performed with a 4.5 MHz probe. 102 B-scan slices are collected. Each B-scan is cropped to 400 × 400 pixels. A depth setting of 200 × 200 mm is used giving a resolution of 0.5 mm/pixel. Figure 3 shows the phantom picture, its inner structure and the reconstructed volume data with our freehand 3D ultrasound reconstruction system.

The second experiment object is an in-vivo liver organ of human, 167 B-scan slices are collected. each B-scan is cropped to 347 × 242 pixels. A depth setting of 177 × 123 mm is used giving a resolution of 0.511945 mm/pixel. Figure 2 shows the bin-filled volume without interpolation and the reconstructed volume using our KR algorithm.

**Figure 4:**
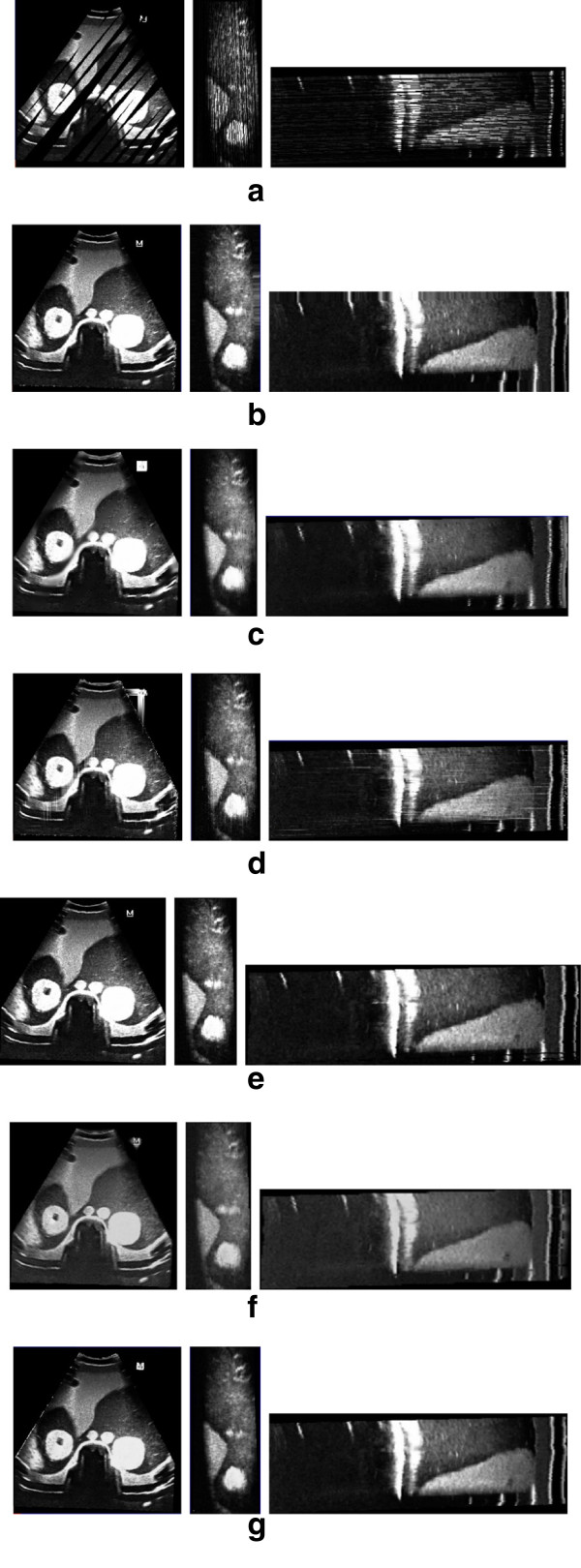
**The coronal, transverse and sagittal reconstructed slice (from left to right) of phantom with different methods. (a)** The bin-filled result. **(b)-(g)** The reconstructed result with **(b)** VNN, **(c)** PNN, **(d)** Spline, **(e)** PT, **(f)** FMM and **(g)** KR.

**Figure 5:**
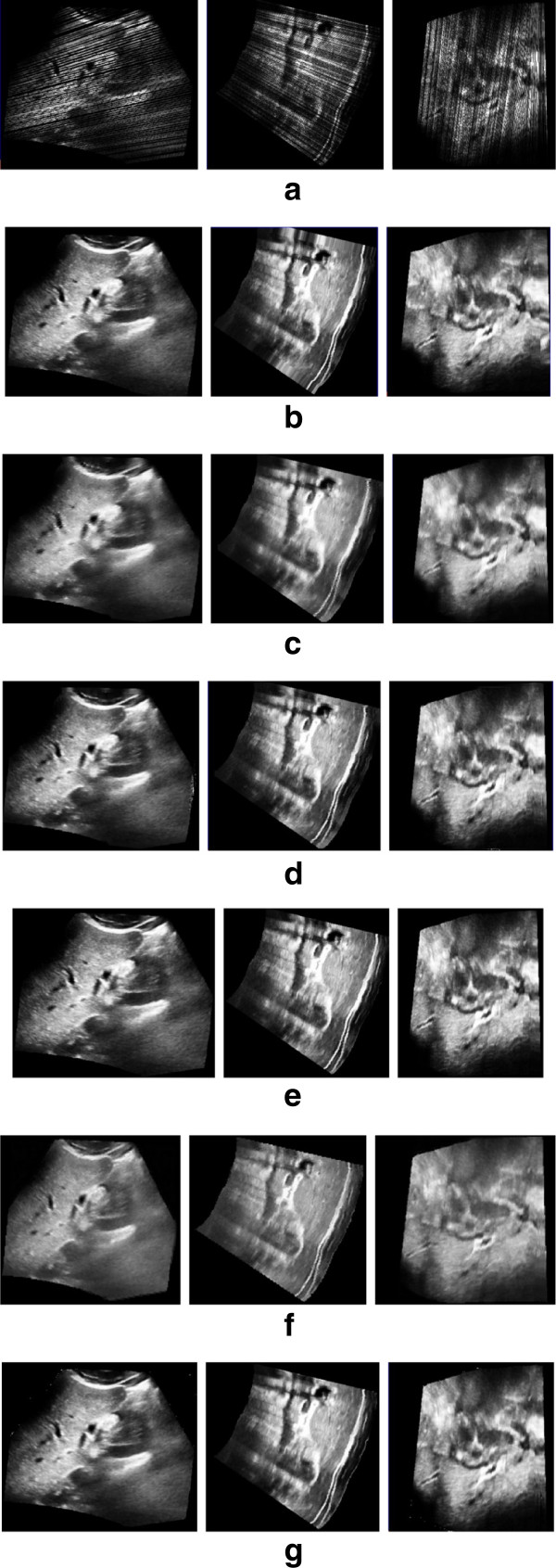
**The coronal, transverse and sagittal reconstructed slice (from left to right) of liver with different methods. (a)** The bin-filled result. **(b)-(g)** The reconstructed result with **(b)** VNN, **(c)** PNN, **(d)** Spline, **(e)** PT, **(f)** FMM and **(g)** KR.

The coronal, sagittal and transverse slice of the phantom and liver using the VNN, PNN, Spline, PT, FMM and KR algorithm are showed in Figure 4 and Figure 5. According to Figure 4(b) and Figure 5(b), the reconstructed slices using the VNN algorithm look sharp and present more texture patterns. However, the anatomical structure is actually distorted due to the misalignments of the pixels, as addressed by [6]. Figure 4(c) and Figure 5(c) show the PNN method causes great blurring as well as relatively large reconstruction error. Overfitting phenomenon is usually unavoidable for the Spline method, as demonstrated in Figure 4(d) and Figure 5(d). Figure 4(e) and Figure 5(e) show the PT method preserve more texture details. However, there are some gaps in the sagittal slice. Figure 4(f) and Figure 5(f) show the recent FMM method performs much better than the classical methods. However, it can’t preserve enough details in the big gap. Compared with the classical methods and the recent FMM method, our KR algorithm has advantages both in recovering missing data with more details and suppressing speckle noises, as demonstrated in Figure 4(g) and Figure 5(g).

### Quantitative results

For the quantitative evaluation, we deliberatly remove a slice from the collected B-Scan slices. Then different reconstruction algorithms are applied to the removed data. Such evaluation method is capable of measuring the ability of a reconstruction algorithm in preserving true intensity values at the locations where a part of original data is removed. A good reconstruction algorithm should interpolate the removed data with values very close to the original data. In this study, root mean square error (RMSE) [28] is used for the image quality measure. It can be described as follow:
14

where *I*_*o*_ is the removed slice from B-scan images, *I*_*r*_ is an image resliced the reconstructed volume with *I*_*o*_’ position. (*x*,*y*) is the coordinate of pixels.

**Figure 6:**
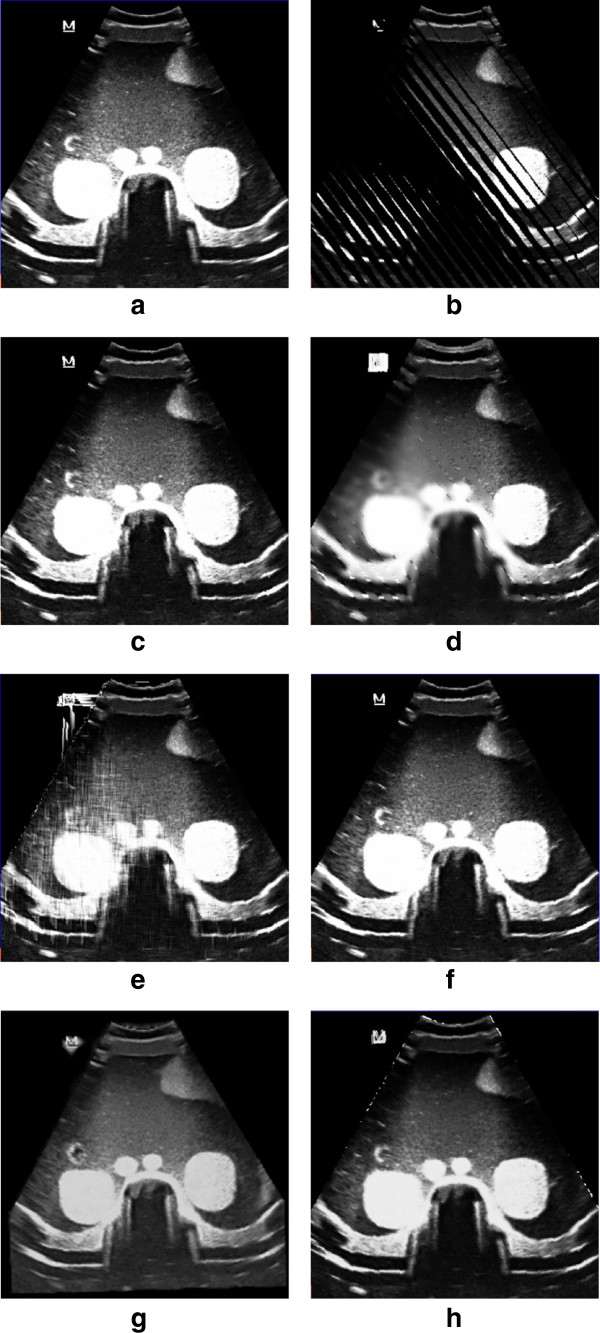
**Comparisons for phantom data with different reconstruction methods. (a)** The original removed phantom image. **(b)** The bin-filled image. **(c)-(h)** The reconstructed result with **(c)** VNN, **(d)** PNN, **(e)** Spline, **(f)** PT, **(g)** FMM and **(h)** KR.

**Figure 7:**
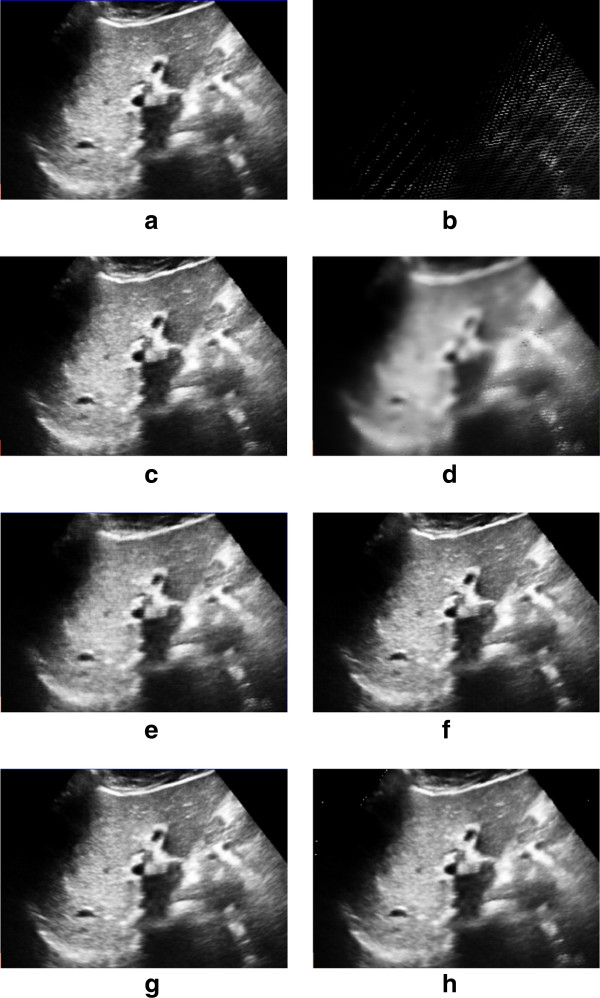
**Comparisons for liver data with different reconstruction methods. (a)** The original removed liver image. **(b)** The bin-filled image. **(c)-(h)** The reconstructed result with **(c)** VNN, **(d)** PNN, **(e)** Spline, **(f)** PT, **(g)** FMM and **(h)** KR.

Table 1 and Table 2 summarize the RMSE of the evaluation tests using the VNN, PNN, Spline, PT, FMM and our KR reconstruction algorithms. The RMSE of our KR algorithm is smallest among these reconstruction mehods. Figure 6 and Figure 7 show that the KR based reconsturcted slice is more close to the original slice.

**Table 1:** **Evaluate the quality of reconstruction of phantom image with RMSE for VNN, PNN, Spline, PT, FMM and KR algorithms**

VNN	PNN	Spline	PT	FMM	KR
14.386524	19.371032	21.436066	13.061580	11.617232	9.335329

**Table 2:** **Evaluate the quality of reconstruction of liver image with RMSE for VNN, PNN, Spline, PT, FMM and KR algorithms**

VNN	PNN	Spline	PT	FMM	KR
9.473721	14.149041	8.307448	11.268841	7.188029	6.682401

### Computation complexity analysis

In order to perform an objective and theoretical estimations to the computation time, the big *O* notation is used to identify the complexity function. Table 3 lists the computational time complexity of VNN, PNN, Spline, PT, FMM and our KR approaches.

**Table 3:** **Computational time complexity for VNN, PNN, Spline, PT, FMM and KR algorithms**

VNN	PNN	Spline	PT	FMM	KR
*O*(*N* · *N*_*p*_)	*O*(*N* · *R* ^3^)	*O*(*N*)		*O*(*N*_*m*_ · *log*(*N*_*m*_) · *R* ^3^)	*O*(*N* · *R* ^6^ · *D*)

*N*_*x*_, *N*_*y*_, *N*_*z*_ are the dimensions of the volume grid in *x*, *y* and *z* direction, *N* ***=*** *N*_*x*_·*N*_*y*_·*N*_*z*_, *N*_*p*_ is the number of B-scans. *N*_*m*_ ***=*** *max*(*N*_*x*_,*N*_*y*_,*N*_*z*_), *R* is the size of cubic region, *d* is the number of dimsentions of the volume, *D* ***=*** *d* ***+***1.

The practical time costs are demonstrated in Table 4 for the phantom test (with a dimension of 424×421×131) and Table 5 for the liver test (with a dimension of 387×338×350). From Table 4 and Table 5, the KR takes more computation time than VNN, PNN, Spline, PT, and FMM algorithms.

**Table 4:** **The running time with VNN, PNN, Spline, PT, FMM and KR in the experiment of phantom**

VNN	PNN	Spline	PT	FMM	KR
1349.371s	1051.297s	55.911s	2528.297s	50.294731s	23366.637s

**Table 5:** **The running time with VNN, PNN, Spline, PT, FMM and KR in the experiment of liver**

VNN	PNN	Spline	PT	FMM	KR
3786.954s	2803.087s	74.583s	7580.02s	60.768321s	38821.058s

## Conclusion

In this paper, A novel freehand 3D ultrasound system based on kernel regression is proposed. A phantom and an in-vivo liver organ of human subject are scanned and reconstructed with this system for experiments. Root mean square error (RMSE) is used for the quantitative evaluation. Both of the qualitative and quantitative experimental results have demonstrated that our method can reconstruct image with less artifacts and higher quality than the classical VNN, PNN, Spline, PT methods and the recent FMM method. Therefore, the proposed kernel regression based reconstruction method is capable of constructing volume data with improved accuracy from irregularly sampled sparse data for freehand 3D ultrasound imaging system. Of course, the practical time cost experiments show it costs more computation time. So the time complexity is required to improve for higher efficiency in freehand 3D ultrasound reconstruction in the future work. More over, in respect to quality, automatic and dynamic bandwidth will be studied for spatial adaptation.

## Abbreviations

2D: Two-dimensional
3D: Three-dimensional
VBM: Voxel-based methods
PBM: Pixel-based methods
FBM: Function-based methods
VNN: Voxel nearest neighbor
PNN: Pixel nearest neighbor
DW: Distance-weighted
FMM: Fast marching method
PT: Probe trajectory
KR: Kernel regression
RBF: Radial basis function
ROI: Region of interest.
